# Age‐Related Clinical Agreement Between Noncycloplegic Autorefraction and Subjective Refraction: A Power‐Vector Analysis

**DOI:** 10.1155/joph/4862472

**Published:** 2026-02-26

**Authors:** Ozlem Candan, Irem Saglam, Gozde Orman, Nurten Unlu, Ayse Burcu

**Affiliations:** ^1^ Department of Ophthalmology, Ankara Training and Research Hospital, Turkish Ministry of Health, Ankara, Turkey, saglik.gov.tr

**Keywords:** age-related refraction changes, autorefraction, power vector analysis, spherical equivalent, subjective refraction

## Abstract

**Purpose:**

To assess the agreement between noncycloplegic autorefraction and subjective refraction across age groups and refractive error types in a large real‐world clinical study using clinically meaningful thresholds for spherical and astigmatic components.

**Methods:**

This retrospective study analyzed 827 eyes from patients aged 7–85 years examined between May 2023 and January 2025. Objective refraction was measured using a KR‐1 autorefractor (Topcon, Tokyo, Japan), followed by standard subjective refinement. Spherical equivalent (SE), cylinder power (CP), and power vectors (J0, J45) were calculated using Fourier vector analysis. Agreement was evaluated using absolute differences and predefined clinical criteria (SE ± 0.50 D; CP ± 0.25 D; axis ≤ 10° when CP ≥ 0.50 D). Comparisons were performed across three age groups (7–21, 22–49, ≥ 50 years) and refractive subgroups.

**Results:**

SE agreement was high across all ages, with 90.8% of eyes within ±0.50 D. J0 demonstrated a progressive shift from with‐the‐rule to against‐the‐rule astigmatism with age, whereas J45 remained near zero. CP differences were generally small but slightly larger in older adults. Axis deviations > 10° occurred in 27.2% of eyes, predominantly when CP was low (mean 0.51 ± 0.42 D), suggesting limited visual impact in most cases.

**Conclusions:**

Noncycloplegic autorefraction has been demonstrated to exhibit a high degree of agreement with subjective refraction for SE in both adolescents and adults. The astigmatic shifts that occurred with age were minimal and had only a limited effect on clinical outcomes. Subjective refinement remains a critical component in the precise determination of axes, particularly in cases of hyperopia, mixed astigmatism, and in eyes of advanced age. Future studies that include hyperopic children and young patients requiring cycloplegia would be valuable in extending these findings to a broader range of pediatric populations.

## 1. Introduction

Refractive errors are the leading cause of visual impairment worldwide, affecting billions of individuals and posing a substantial public health burden [1, 2]. Accurate refractive assessment is essential for optimizing visual outcomes, prescribing spectacle correction, and provides reliable data for population‐based studies. Autorefraction is a rapid and standardized objective method that is widely used in clinical and screening settings. However, its accuracy may be influenced by accommodation, pupil size, fixation, instrument design, and neural adaptation [3]. Subjective refraction remains the clinical gold standard, incorporating patient‐perceived clarity and binocular balance.

A substantial body of research has examined the correlation between objective and subjective refraction in specific age groups or refractive error subpopulations. Kozlov et al. [4] reported that subjective refraction yielded less minus power in myopes and less plus power in hyperopes compared with autorefraction in healthy young adults. In adult myopes, disparities between cycloplegic and noncycloplegic refraction have been associated with accommodative effects [5]. In children and adolescents, particularly those with hyperopia, cycloplegia significantly impacts measured refraction relative to noncycloplegic values [6]. Taken together, agreement was influenced by age, refractive error type, accommodative status, and testing methodology.

Despite these contributions, large real‐world datasets spanning from childhood to older adulthood under routine clinical conditions remain limited. A considerable body of research has focused on either pediatric or adult samples, or has employed research protocols that deviate from standard clinical practice [7, 8]. Moreover, there are a limited number of reports that have examined both spherical and vector components across various refractive groups utilizing well‐established power‐vector methodologies [9, 10]. Furthermore, the clinical significance of predefined agreement thresholds and axis deviation in astigmatism has been addressed only to a limited extent in general ophthalmic settings [11, 12].

The present study evaluates the agreement between noncycloplegic autorefraction and subjective refraction across three age groups representing different levels of accommodative capacity, with stratification by refractive error type and sex. The purpose of this study was to evaluate the agreement between noncycloplegic autorefraction and subjective refraction across different age groups and refractive error types under routine clinical conditions.

## 2. Methods

### 2.1. Study Design and Participants

This retrospective observational study included 827 eyes from 827 patients who underwent routine ophthalmic examinations at a tertiary outpatient clinic between May 1, 2023, and January 1, 2025. Data were extracted from electronic medical records. Eligible participants were ≥ 7 years, literate, and had best‐corrected visual acuity (BCVA) of 6/6 in each eye, ensuring reliable response. Objective (autorefraction) and subjective refraction had to be performed during the same clinical visit.

Exclusion criteria included a history of ocular surgery, keratoconus or other corneal ectasia, corneal opacity, lens opacity > grade 1, retinal or optic nerve disease affecting refraction, narrow angles, anterior segment anomalies, chronic ocular inflammation, prior strabismus or amblyopia treatment, and incomplete data. In order to account for age‐related changes in accommodative amplitude, participants were divided into three predefined age groups: 7–21 years (high accommodation), 22–49 years (moderate accommodation), and ≥ 50 years (minimal accommodation). All measurements were obtained using the same auto kerato‐refractometer (KR‐1; Topcon Corporation, Tokyo, Japan). The instrument was operated in accordance with the manufacturer’s standard maintenance and calibration procedures, under standardized conditions by two ophthalmologists (O.C. and I.S.) to ensure procedural consistency.

The study was based on routine noncycloplegic clinical data; therefore, children with suspected latent hyperopia or accommodative anomalies requiring cycloplegic refraction were excluded from the analysis. As a result, hyperopic children were not represented in the youngest age group. This methodological decision was made to minimize accommodative confounding in noncycloplegic measurements and should be considered when interpreting age‐related refractive comparisons.

### 2.2. Objective Refraction Procedure

Objective refraction was performed using an automated kerato‐refractometer (KR‐1; Topcon Corporation, Tokyo, Japan) in accordance with standardized conditions. Measurements were obtained monocularly, with the other eye occluded. Three consecutive measurements were taken for each eye, and the mean of these readings was used in the analysis to ensure stability and reduce measurement noise. Any reading that differed from the median by more than ±1.0 D was considered an outlier and excluded; the eye was then remeasured.

The instrument’s internal fogging mechanism was activated to minimize accommodative stimulation and the patients fixated on a high‐contrast distance target equivalent to 6 m. Examinations were conducted in a dimly lit room (approximately 80 lux) to promote consistent pupil size and accommodative relaxation. The device automatically optimized pupil alignment throughout the procedure.

Cycloplegia was not administered because the study utilized routine clinical data and cycloplegic refraction was only reserved for clinically suspected latent hyperopia or accommodative anomalies. Such cases were excluded from the analysis, resulting in a cohort that represented typical noncycloplegic clinical practice. This limitation should be considered when interpreting results in younger hyperopes.

### 2.3. Subjective Refraction Protocol

Immediately after autorefraction, subjective refraction was performed by the same two ophthalmologists using a phoropter and a Snellen chart positioned at 6 m under standardized photopic illumination (approximately 300 lux). The subjective refraction procedure was initiated using a standardized fogging technique to relax accommodation, followed by a gradual reduction of plus power to determine the maximum plus power for best visual acuity (MPBVA). The spherical equivalent (SE) obtained from autorefraction was used as an initial reference estimate, not as the primary starting value. The duochrome test was performed as a secondary check to confirm the neutrality of the focus plane.

Astigmatic refinement was conducted using the Jackson cross‐cylinder (±0.25 D) with axis adjustments in 5° steps, refined to 2.5° when cylinder ≥ 1.50 D. Cylinder power (CP) was modified in 0.25 D increments. For eyes with higher cylinders (≥ 1.50 D), fine‐tuning steps of 2.5° were employed to minimize axis error. Confirmation was achieved with endpoint reversal (over‐minus/over‐plus check), and duochrome test was used when appropriate. In cases of cylinder > 3.00 D, axis refinements were performed with greater caution, with repeated JCC checks to avoid over‐correction.

Each eye was tested monocularly before final binocular balancing to ensure comparable accommodation between the two eyes once monocular best visual acuity had been achieved.

In patients receiving their first spectacle correction, the recorded refractive value reflected the final subjective refraction rather than the dispensed prescription, which may be modified to facilitate visual adaptation. This distinction enabled the analysis of pure refractive agreement, independent of wearer experience. In cases where discrepancies between examiners exceeded ±0.25 D or visual acuity endpoints were inconsistent, a senior examiner performed repeat refraction to confirm agreement. In order to ensure reproducibility, all procedures were conducted under identical room lighting and target conditions across participants.

### 2.4. Calculation of SE, Power Vectors, and Differences Between Objective and Subjective Refraction

Refractive error was expressed using the power vector notation. The SE, the Jackson cross‐cylinder components (J0, J45), CP were calculated from the sphere (S), the cylinder (C), and the axis (A) according to the following formulas [9, 10]:

(1) SE=S+C2,a=A∗2π180,J0=−12∗C∗cos  2a,J45=−12∗C∗sin  2a,CP=2∗J02+J452.

The differences between these two refraction values were calculated by subtracting the subjective refraction values from the objective refraction values (i.e., objective minus subjective). For each eye, the absolute differences between objective and subjective refraction were calculated for both SE and CP; the absolute differences (ΔADs) were reported as the primary measure, since they are clinically intuitive and directly interpretable in diopters.

(2) ΔAD=Xauto−Xsubj.

Furthermore, the symmetric percentage difference (SPD) was calculated as a secondary measure to complement the absolute difference. This approach normalizes the discrepancy by combining the magnitudes of both measurements, thus enabling proportional comparisons across eyes with different refractive powers. The primary function of this approach is to prevent the underestimation of differences in eyes with low dioptric values, thereby providing a more balanced assessment of agreement across the refractive range.

(3) SPD=100∗Xauto−XsubjXauto+Xsubj/2.

Signed difference (ΔSD) was also specifically calculated for J0 and J45 components, as they preserve the directional information of astigmatic bias in addition to its magnitude.

(4) ΔSD=Xauto−Xsubj.

Moreover, in astigmatic eyes, CP, J0, and J45 values from autorefraction and keratometry were compared to evaluate corneal versus internal astigmatism. The absolute and signed differences were then calculated by subtracting the keratometry values from the autorefraction values.

For eyes with astigmatism accompanied by myopia or hyperopia, the difference vector (DV), representing the Euclidean distance in power vector space, was calculated using the following formula to express the total refractive difference as a single metric:

(5) DV=SEauto−SEsubj2+J0auto−J0subj2+J45auto−J45subj2.

In order to facilitate clinically meaningful interpretation of vector differences in astigmatism, J0 and J45 discrepancies were additionally converted to an equivalent axis shift (ΔAX) using the following formula:

(6) ΔAX=180/π∗J0auto−J0subj2+J45auto−J45subj2Csubj.

### 2.5. Agreement Analysis and Statistical Methods

For the purposes of statistical analysis, the right eye of each participant was included, thus ensuring that intereye correlation was eliminated from the study. The statistical analysis was conducted using Statsmodels 0.14.4 in Python 3.13.7. Normality of continuous variables was assessed with the Shapiro–Wilk test and Q–Q plots. Descriptive data are presented as mean ± standard deviation (SD) for normally distributed variables and median (interquartile range, IQR) for skewed distributions. The agreement between autorefraction and subjective refraction was evaluated using both Bland–Altman analysis and clinically defined equivalence thresholds.

For each refractive component (SE, J0, and J45), the mean bias ± 95% limits of agreement (LoA) were calculated (mean difference ± 1.96 × SD of differences). Bland–Altman plots were then stratified by both age group and refractive error type (myopia, hyperopia, and astigmatism of at least 0.50 diopters). Furthermore, the proportion of eyes that met predefined clinical agreement thresholds was reported as a measure of interchangeability: The SE is to be within ±0.50 D, the CP is to be within ±0.25 D, and the cylinder axis is to be within ±10° (for eyes with cylinder ≥ 0.50 D).

To translate vector differences into clinically interpretable axis deviation, J0/J45 discrepancies were converted to an equivalent axis shift (ΔAX) using the following formula:

(7) ΔAX=180/π∗J0auto−J0subj2+J45auto−J45subj2/Csubj.

The ΔAX formula was used to convert vector differences in astigmatism (J0 and J45) into an equivalent axis shift in degrees, thereby providing a clinically interpretable measure of axis change at the patient’s CP. This enabled the assessment of whether astigmatic discrepancies were within clinically acceptable limits for axis alignment. Axis deviation metrics (ΔAX) were analyzed for eyes with subjective cylinder ≥ 0.75 D to ensure clinical relevance and avoid inflated values in low‐cylinder eyes.

Outliers (> ±3 SD from the group mean) were subjected to scrutiny for the presence of transcription or data‐entry errors, and subsequently excluded from sensitivity analyses. Robust statistics (median ± IQR) were recalculated post‐exclusion in order to verify the stability of agreement estimates. The Kruskal–Wallis test was performed to ascertain the differences between the three groups, while the Mann–Whitney *U* test was used to determine the difference between the two groups. To address the issue of false‐positive risks associated with multiple comparisons, a post hoc analysis with Bonferroni corrections was conducted. Statistical significance was determined by establishing a *p* value less than 0.05.

## 3. Results

A total of 827 participants were included in the analysis, of whom 460 (55.6%) were female. Group 1 (ages 7–21) included 214 participants (59.3% female), Group 2 (ages 22–49) included 353 participants (60.0% female), and Group 3 (ages ≥ 50) included 260 participants (47.0% female).

### 3.1. Differences Between Objective and Subjective Refraction in the Overall Study Group

**FIGURE 1:**
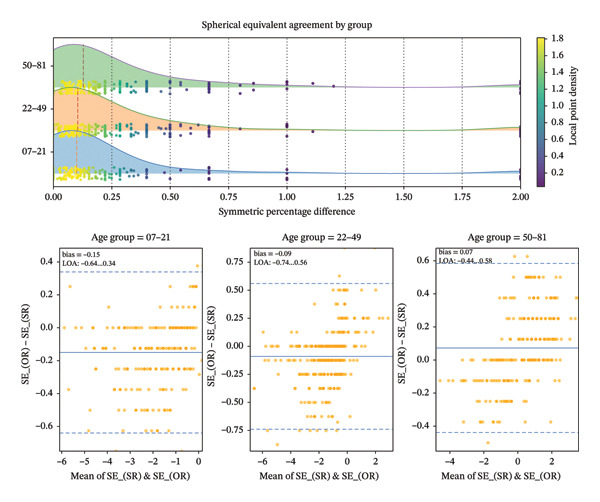
Distribution of symmetric percentage differences (SPD) in spherical equivalent (SE) across age groups and Bland–Altman plot showing the differences in SE between the objective and subjective refraction.

**FIGURE 2:**
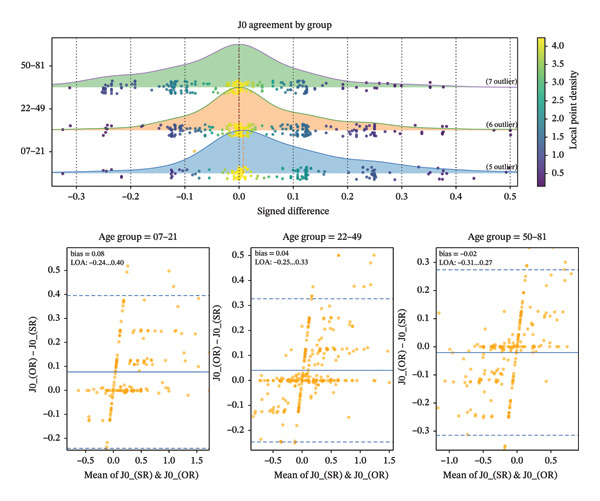
Distribution of *J*_0_ differences across age groups and Bland–Altman plot showing the differences in *J*_0_ between the objective and subjective refraction.

**FIGURE 3:**
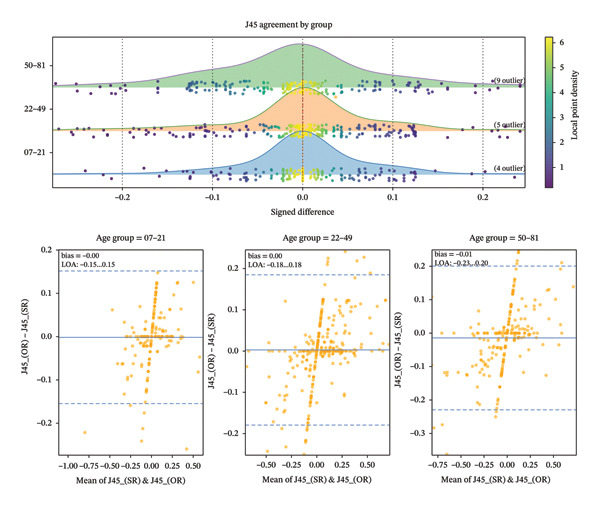
Distribution of *J*_45_ differences across age groups and Bland–Altman plot showing the differences in *J*_45_ between the objective and subjective refraction.

The mean absolute SE difference between autorefraction and subjective refraction was minimal across age categories (7–21 years: 0.21 ± 0.20 D; 22–49 years: 0.22 ± 0.27 D; ≥ 50 years: 0.20 ± 0.18 D; *p* > 0.05). The graphical distributions and Bland–Altman agreement analyses for SE, J0, and J45 across age groups are illustrated in Figures 1, 2, and 3. Moreover, detailed descriptive statistics and age‐group comparisons for spherical and vector components are presented in Table 1.

**TABLE 1:** Differences between objective and subjective refraction by age group.

Variable	07–21 (*N* = 214) (F/M = 126/88)		22–49 (*N* = 353) (F/M = 212/141)		50–81 (*N* = 260) (F/M = 122/138)		*p*
	Mean ± SD	Median (min/max)	Mean ± SD	Median (min/max)	Mean ± SD	Median (min/max)	
Age (years)	15.88 ± 3.70	17.00 (7.00/21.00)	33.39 ± 8.58	31.00 (22.00/49.00)	59.82 ± 7.08	59.00 (50.00/81.00)	**p** < 0.001
K1	42.95 ± 1.15	43.00 (40.25/48.50)	43.06 ± 1.22	43.00 (38.00/46.75)	43.27 ± 1.43	43.25 (40.75/56.75)	**0.039**
K2	43.75 ± 1.12	43.75 (41.25/48.25)	43.75 ± 1.17	43.75 (38.25/46.75)	43.77 ± 1.60	43.75 (41.25/62.25)	0.628
SE_(OR)	−2.01 ± 1.55	−1.75 (−7.00/3.62)	−1.46 ± 1.90	−1.25 (−9.75/6.50)	0.28 ± 1.84	0.62 (−10.38/5.50)	**p** < 0.001
J0_(OR)	0.31 ± 0.50	0.24 (−1.62/2.23)	0.14 ± 0.50	0.10 (−1.32/2.36)	−0.12 ± 0.48	−0.11 (−2.38/2.97)	**p** < 0.001
J45_(OR)	−0.00 ± 0.26	0.00 (−1.62/0.94)	0.04 ± 0.27	0.03 (−1.34/0.93)	−0.03 ± 0.31	0.00 (−1.00/1.97)	**0.002**
CP_(OR)	0.93 ± 0.90	0.75 (0.00/4.50)	0.88 ± 0.77	0.75 (0.00/4.75)	0.88 ± 0.76	0.75 (0.00/6.25)	0.905
SE_(SR)	−1.86 ± 1.47	−1.50 (−7.00/3.38)	−1.37 ± 1.78	−1.00 (−9.50/5.50)	0.20 ± 1.70	0.50 (−10.00/4.88)	**p** < 0.001
J0_(SR)	0.23 ± 0.42	0.10 (−1.30/1.72)	0.10 ± 0.41	0.00 (−0.98/2.09)	−0.10 ± 0.38	0.00 (−1.75/2.11)	**p** < 0.001
J45_(SR)	−0.00 ± 0.23	0.00 (−1.50/0.75)	0.04 ± 0.22	0.00 (−1.11/0.86)	−0.01 ± 0.25	0.00 (−0.87/1.48)	**0.001**
CP_(SR)	0.68 ± 0.82	0.50 (0.00/3.50)	0.66 ± 0.69	0.50 (0.00/4.25)	0.63 ± 0.69	0.50 (0.00/4.50)	0.722
ΔAD between SE_(OR) and SE_(SR)	0.21 ± 0.20	0.12 (0.00/1.00)	0.22 ± 0.27	0.12 (0.00/2.00)	0.20 ± 0.18	0.12 (0.00/0.88)	0.688
SPD between SE_(OR) and SE_(SR)	22.08 ± 40.22	10.08 (0.00/200.00)	25.88 ± 44.33	10.53 (0.00/200.00)	27.13 ± 41.96	12.92 (0.00/200.00)	0.137
ΔSD between J0_(OR) and J0_(SR)	0.08 ± 0.16	0.01 (−0.38/1.00)	0.04 ± 0.15	0.00 (−0.62/0.70)	−0.02 ± 0.15	0.00 (−0.62/0.86)	**p** < 0.001
ΔSD between J45_(OR) and J45_(SR)	−0.00 ± 0.08	0.00 (−0.35/0.25)	0.00 ± 0.09	0.00 (−0.30/0.62)	−0.01 ± 0.11	0.00 (−0.43/0.49)	**0.021**
ΔAD between CP_(OR) and CP_(SR)	0.25 ± 0.30	0.25 (0.00/2.00)	0.23 ± 0.27	0.25 (0.00/1.50)	0.26 ± 0.26	0.25 (0.00/1.75)	**0.031**
SPD between CP_(OR) and CP_(SR)	74.23 ± 88.79	25.00 (0.00/200.00)	65.63 ± 83.18	28.57 (0.00/200.00)	78.84 ± 88.31	28.57 (0.00/200.00)	0.171
DV between OR and SR	0.26 ± 0.24	0.18 (0.00/1.42)	0.37 ± 2.13	0.18 (0.00/40.00)	0.26 ± 0.20	0.18 (0.00/1.24)	0.514
ΔSD between J0_(OR) and J0_(KR)	0.06 ± 0.47	0.00 (−0.98/1.94)	−0.02 ± 0.43	0.00 (−1.75/2.14)	−0.12 ± 0.56	−0.11 (−3.00/2.00)	**0.003**
ΔSD between J45_(OR) and J45_(KR)	−0.02 ± 0.23	0.00 (−1.26/1.06)	0.01 ± 0.27	0.00 (−1.48/1.11)	−0.04 ± 0.34	0.00 (−1.30/1.10)	0.306
ΔAD between CP_(OR) and CP_(KR)	0.61 ± 0.57	0.50 (0.00/2.75)	0.50 ± 0.51	0.50 (0.00/2.75)	0.51 ± 0.46	0.50 (0.00/3.50)	0.186
SPD between CP_(OR) and CP_(KR)	72.38 ± 59.05	66.67 (0.00/200.00)	65.41 ± 58.81	66.66 (0.00/200.00)	67.70 ± 53.54	66.66 (0.00/200.00)	0.332
DV between OR and KR	0.52 ± 0.53	0.35 (0.00/3.18)	0.50 ± 0.52	0.35 (0.00/3.18)	0.73 ± 0.61	0.71 (0.00/4.24)	**p** < 0.001

*Note:* J0 and J45 = Jackson cross‐cylinder components, ΔAD = absolute differences, ΔSD = signed differences, DV = difference vector (the Euclidean distance in power vector space), KR = values derived from keratometric readings. Data are presented as mean ± standard deviation and median (minimum–maximum) values, as appropriate. *p* values indicate between‐group comparisons using the Kruskal–Wallis test. Post hoc pairwise comparisons were conducted using Bonferroni correction. Statistical significance was defined as *p* < 0.05. Bold values indicate statistically significant results.

Abbreviations: CP, cylinder power; OR, Objective refraction; SD, standard deviation; SE, spherical equivalent; SPD, symmetric percentage difference; SR, subjective refraction.

The mean absolute cylinder differences exhibited significant variation by age group (*p* = 0.031), with post hoc analysis indicating that the ≥ 50‐year group demonstrated significantly larger differences compared with the 22–49 years group (7–21 years: 0.25 ± 0.30 D; 22–49 years: 0.23 ± 0.27 D; ≥ 50 years: 0.26 ± 0.26 D).

Signed J0 differences differed significantly across all three age groups, although the magnitude of these differences was minimal (0.08 ± 0.16 D; 0.04 ± 0.15 D; −0.02 ± 0.15 D; *p* < 0.001).

Furthermore, a statistically significant difference was identified in the signed J45 differences, as evidenced by post hoc analysis, which revealed that the ≥ 50‐year group exhibited a distinct pattern compared to the younger groups (*p* = 0.021). However, across all age groups, the numerical values remained close to zero.

### 3.2. Gender Comparison

**FIGURE 4:**
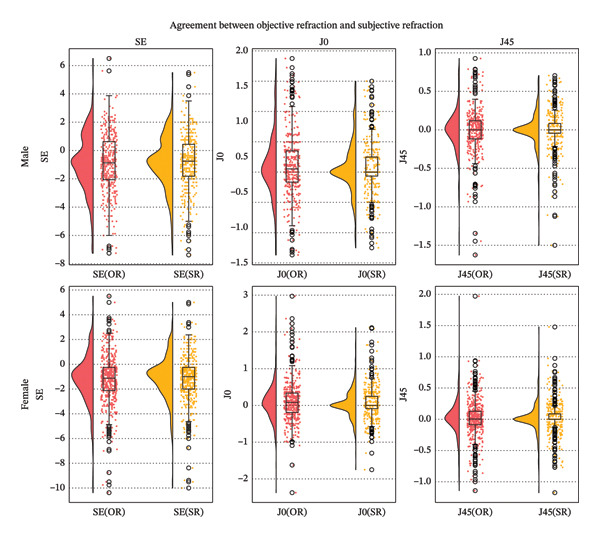
Distribution of objective and subjective refraction values (SE, *J*_0_, and *J*_45_) by sex.

No clinically meaningful differences were observed between autorefraction and subjective refraction according to gender. Men were slightly older (38.9 ± 19.0 versus 35.8 ± 17.7 years; *p* = 0.017) and showed marginally lower myopia in both objective and subjective SE measurements (*p* = 0.042 and *p* = 0.047, respectively), whereas absolute SE differences were similar between genders. A mild increase in the discrepancy between total and corneal astigmatism was observed in males (DV: 0.63 ± 0.56 versus 0.53 ± 0.56 D; *p* = 0.003), indicating a slightly greater internal–corneal component; however, the magnitude was small and unlikely to affect clinical refraction. A summary of gender‐related agreement metrics is provided in Table 2 and Figure 4.

**TABLE 2:** Differences between objective and subjective refraction by gender.

Variable	Female (*N* = 460)		Male (*N* = 367)		*p*
	Mean ± SD	Median (min/max)	Mean ± SD	Median (min/max)	
Age (years)	35.80 ± 17.67	31.00 (7.00/81.00)	38.87 ± 18.99	35.00 (8.00/81.00)	**0.017**
K1	43.10 ± 1.38	43.00 (38.00/56.75)	43.10 ± 1.14	43.00 (40.50/46.75)	0.787
K2	43.75 ± 1.47	43.75 (38.25/62.25)	43.77 ± 1.08	43.75 (41.25/47.00)	0.463
SE_(OR)	−1.19 ± 1.99	−1.12 (−10.38/5.50)	−0.89 ± 2.05	−0.88 (−7.25/6.50)	**0.042**
J0_(OR)	0.10 ± 0.52	0.08 (−2.38/2.97)	0.10 ± 0.52	0.05 (−1.37/1.88)	0.948
J45_(OR)	0.02 ± 0.28	0.00 (−1.14/1.97)	−0.01 ± 0.29	0.00 (−1.62/0.93)	0.089
CP_(OR)	0.88 ± 0.81	0.75 (0.00/6.25)	0.91 ± 0.79	0.75 (0.00/4.50)	0.672
SE_(SR)	−1.13 ± 1.84	−1.00 (−10.00/5.00)	−0.85 ± 1.92	−0.75 (−7.38/5.50)	**0.047**
J0_(SR)	0.06 ± 0.42	0.00 (−1.75/2.11)	0.08 ± 0.43	0.00 (−1.25/1.50)	0.577
J45_(SR)	0.02 ± 0.23	0.00 (−1.17/1.48)	−0.00 ± 0.24	0.00 (−1.50/0.70)	0.144
CP_(SR)	0.63 ± 0.73	0.50 (0.00/4.50)	0.68 ± 0.72	0.50 (0.00/3.50)	0.359
ΔAD between SE_(OR) and SE_(SR)	0.21 ± 0.22	0.12 (0.00/2.00)	0.21 ± 0.24	0.12 (0.00/2.00)	0.820
SPD between SE_(OR) and SE_(SR)	27.18 ± 46.51	10.53 (0.00/200.00)	22.91 ± 36.93	11.76 (0.00/200.00)	0.794
ΔSD between J0_(OR) and J0_(SR)	0.04 ± 0.16	0.00 (−0.62/1.00)	0.02 ± 0.16	0.00 (−0.62/0.62)	0.504
ΔSD between J45_(OR) and J45_(SR)	−0.00 ± 0.10	0.00 (−0.43/0.62)	−0.01 ± 0.09	0.00 (−0.32/0.35)	0.109
ΔAD between CP_(OR) and CP_(SR)	0.24 ± 0.28	0.25 (0.00/2.00)	0.24 ± 0.26	0.25 (0.00/1.25)	0.802
SPD between CP_(OR) and CP_(SR)	73.69 ± 87.22	28.57 (0.00/200.00)	70.08 ± 85.42	28.57 (0.00/200.00)	0.610
DV between OR and SR	0.26 ± 0.24	0.18 (0.00/2.00)	0.37 ± 2.09	0.18 (0.00/40.00)	0.888
ΔSD between J0_(OR) and J0_(KR)	−0.05 ± 0.47	−0.02 (−3.00/2.14)	−0.02 ± 0.51	−0.02 (−2.00/2.00)	0.826
ΔSD between J45_(OR) and J45_(KR)	−0.00 ± 0.27	0.00 (−1.48/1.10)	−0.02 ± 0.31	0.00 (−1.30/1.11)	0.114
ΔAD between CP_(OR) and CP_(KR)	0.52 ± 0.52	0.50 (0.00/3.50)	0.55 ± 0.51	0.50 (0.00/2.75)	0.278
SPD between CP_(OR) and CP_(KR)	67.55 ± 57.24	66.66 (0.00/200.00)	68.43 ± 57.38	66.66 (0.00/200.00)	0.866
DV between OR and KR	0.53 ± 0.56	0.35 (0.00/4.24)	0.63 ± 0.56	0.53 (0.00/3.01)	**0.003**

*Note:* J0 and J45 =  Jackson cross‐cylinder components, ΔAD = absolute differences, ΔSD = signed differences, DV = difference vector (the Euclidean distance in power vector space), KR = values derived from keratometric readings. Data are presented as mean ± standard deviation and median (minimum–maximum) values, as appropriate. *p* values indicate between‐group comparisons using the Kruskal–Wallis test. Post hoc pairwise comparisons were conducted using Bonferroni correction. Statistical significance was defined as *p* < 0.05. Bold values indicate statistically significant results.

Abbreviations: CP, cylinder power; OR, Objective refraction; SD, standard deviation; SE, spherical equivalent; SPD, symmetric percentage difference; SR, subjective refraction.

### 3.3. Myopia Subgroup

SE agreement remained high among myopic eyes across all age groups, with mean absolute differences of 0.20 ± 0.20 D (7–21 years), 0.21 ± 0.26 D (22–49 years), and 0.21 ± 0.21 D (≥ 50 years) (*p* > 0.05). These findings suggest that accommodation did not significantly impact objective‐subjective agreement in patients with myopia.

### 3.4. Hyperopia Subgroup

No hyperopic patients were included in the 7–21 years group due to the exclusion of latent hyperopes requiring cycloplegia. Among adults, SE agreement exhibited slightly greater variability in the 22–49 years group (ΔAD: 0.33 ± 0.40 D) compared with the ≥ 50 years group (ΔAD: 0.20 ± 0.16 D), although the difference was not statistically significant (*p* > 0.05). Agreement results by refractive error type are summarized in Table 3.

**TABLE 3:** Differences between objective and subjective refraction by age group in myopic and hyperopic eyes.

**Myopia**	**07–21 (*N* = 188) (F/M = 108/80)**		**22–49 (*N* = 274) (F/M = 162/112)**		**50–81 (*N* = 89) (F/M = 49/40)**		**p**
**Variable**	**Mean ± SD**	**Median (min/max)**	**Mean ± SD**	**Median (min/max)**	**Mean ± SD**	**Median (min/max)**	

Age (years)	15.65 ± 3.76	16.00 (7.00/21.00)	31.88 ± 7.94	30.00 (22.00/49.00)	60.13 ± 8.45	58.00 (50.00/81.00)	**p** < 0.001
SE_(OR)	−2.28 ± 1.42	−2.00 (−7.00/0.38)	−2.04 ± 1.61	−1.62 (−9.75/1.00)	−1.68 ± 1.50	−1.38 (−10.38/0.12)	**p** < 0.001
SE_(SR)	−2.11 ± 1.39	−1.75 (−7.00/‐0.50)	−1.91 ± 1.53	−1.50 (−9.50/‐0.50)	−1.60 ± 1.38	−1.25 (−10.00/‐0.50)	**0.002**
ΔAD between SE_(OR) and SE_(SR)	0.20 ± 0.20	0.12 (0.00/1.00)	0.21 ± 0.26	0.12 (0.00/2.00)	0.21 ± 0.21	0.12 (0.00/0.88)	0.490
SPD between SE_(OR) and SE_(SR)	12.56 ± 18.49	8.00 (0.00/200.00)	16.23 ± 28.17	8.51 (0.00/200.00)	25.69 ± 43.46	11.76 (0.00/200.00)	0.076

**Hyperopia**			**22–49 (*N* = 30) (F/M = 21/9)**		**50–81 (*N* = 134) (F/M = 52/82)**		**p**
**Variable**	**Mean ± SD**	**Median (min/max)**	**Mean ± SD**	**Median (min/max)**	**Mean ± SD**	**Median (min/max)**	

Age (years)	—	—	41.43 ± 7.66	44.00 (22.00/49.00)	60.10 ± 6.46	59.00 (50.00/80.00)	**p** < 0.001
SE_(OR)	—	—	1.82 ± 1.54	1.25 (−0.12/6.50)	1.61 ± 0.84	1.50 (0.25/5.50)	0.818
SE_(SR)	—	—	1.67 ± 1.49	1.12 (0.50/5.50)	1.45 ± 0.79	1.25 (0.50/4.88)	0.238
ΔAD between SE_(OR) and SE_(SR)	—	—	0.33 ± 0.40	0.25 (0.00/1.75)	0.20 ± 0.16	0.12 (0.00/0.62)	0.546
SPD between SE_(OR) and SE_(SR)	—	—	24.14 ± 36.86	17.42 (0.00/200.00)	16.15 ± 17.04	11.76 (0.00/85.71)	0.405

*Note:* F =female, M = male, ΔAD = absolute differences, data are presented as mean ± standard deviation and median (minimum–maximum) values, as appropriate. *p* values indicate between‐group comparisons using the Kruskal–Wallis test or Mann–Whitney *U* test. Post hoc pairwise comparisons were conducted using Bonferroni correction. Statistical significance was defined as *p* < 0.05. Bold values indicate statistically significant results.

Abbreviations: OR, objective refraction; SD, standard deviation; SE, spherical equivalent; SPD, symmetric percentage difference; SR, subjective refraction.

### 3.5. Astigmatic Subgroup

Eyes with pure astigmatism demonstrated minimal vector differences across age groups, with mean J0 and J45 values near zero (*p* > 0.05) and negligible differences in CP (≤ 0.17 D). The axis deviation per 1.00 D of cylinder did not differ significantly between age groups (*p* > 0.05), indicating comparable axis agreement across the cohort.

In mixed astigmatism, a statistically significant age‐related shift in J0 was observed (*p* < 0.001), consistent with the transition from with‐the‐rule to against‐the‐rule astigmatism with age. In contrast, J45 and cylinder magnitude showed no significant age‐dependent differences (*p* > 0.05).

Differences between total and corneal astigmatism increased with age in mixed‐astigmatic eyes, as shown by significant differences in signed J0 and J45 comparisons (*p* <0.001 and *p* = 0.015, respectively). The DV between autorefraction and keratometry was significantly higher in the ≥ 50 years group (*p* = 0.002). Table 4 presents the detailed vector‐based analyses.

**TABLE 4:** Differences between objective and subjective refraction by age group in pure and mixed astigmatism.

**Eyes with pure astigmatism**	**07–21 (*N* = 14) (F/M = 10/4)**		**22–49 (*N* = 34) (F/M = 20/14)**		**50–81 (*N* = 21) (F/M = 13/8)**		**p**
**Variable**	**Mean ± SD**	**Median (min/max)**	**Mean ± SD**	**Median (min/max)**	**Mean ± SD**	**Median (min/max)**	

Age (years)	17.14 ± 2.82	17.50 (13.00/21.00)	37.56 ± 8.46	41.00 (23.00/49.00)	58.14 ± 5.42	57.00 (51.00/68.00)	**< 0.001**
ΔSD between J0_(OR) and J0_(SR)	−0.01 ± 0.12	0.00 (−0.32/0.12)	0.01 ± 0.09	0.00 (−0.18/0.23)	−0.02 ± 0.06	−0.01 (−0.12/0.14)	0.229
ΔSD between J45_(OR) and J45_(SR)	0.01 ± 0.07	0.00 (−0.13/0.19)	0.01 ± 0.05	0.00 (−0.08/0.18)	−0.01 ± 0.15	−0.00 (−0.36/0.49)	0.287
ΔAD between CP_(OR) and CP_(SR)	0.14 ± 0.23	0.00 (0.00/0.75)	0.13 ± 0.15	0.00 (0.00/0.50)	0.17 ± 0.28	0.00 (0.00/1.00)	0.703
SPD between CP_(OR) and CP_(SR)	8.84 ± 13.88	0.00 (0.00/40.00)	13.85 ± 17.34	0.00 (0.00/50.00)	13.33 ± 19.93	0.00 (0.00/66.66)	0.466
DV between OR and SR	0.31 ± 0.26	0.25 (0.00/0.89)	0.26 ± 0.16	0.25 (0.00/0.75)	0.23 ± 0.18	0.25 (0.00/0.56)	0.670
ΔAX@1D between OR and SR	4.60 ± 6.61	1.00 (0.00/21.49)	4.06 ± 4.10	1.50 (0.00/14.36)	5.31 ± 7.69	1.00 (0.00/28.65)	0.761
ΔSD between J0_(OR) and J0_(KR)	−0.02 ± 0.47	0.00 (−0.58/1.21)	−0.02 ± 0.48	0.00 (−0.84/2.14)	−0.17 ± 0.24	−0.12 (−0.57/0.20)	0.236
ΔSD between J45_(OR) and J45_(KR)	0.02 ± 0.18	0.00 (−0.30/0.31)	0.05 ± 0.23	0.00 (−0.43/0.93)	−0.20 ± 0.39	−0.10 (−1.19/0.37)	**0.038**
ΔAD between CP_(OR) and CP_(KR)	0.62 ± 0.69	0.37 (0.00/2.50)	0.24 ± 0.24	0.25 (0.00/0.75)	0.45 ± 0.37	0.25 (0.00/1.50)	0.146
SPD between CP_(OR) and CP_(KR)	61.70 ± 54.88	53.33 (0.00/200.00)	35.25 ± 37.40	28.57 (0.00/120.00)	52.61 ± 35.35	40.00 (0.00/133.33)	0.181
DV between OR and KR	0.49 ± 0.50	0.35 (0.00/1.77)	0.41 ± 0.63	0.18 (0.00/3.18)	0.57 ± 0.46	0.53 (0.00/1.77)	0.214

**Eyes with mixed astigmatism**	**07–21 (*N* = 93) (F/M = 48/45)**		**22–49 (*N* = 153) (F/M = 89/64)**		**50–81 (*N* = 112) (F/M = 55/57)**		**p**
**Variable**	**Mean ± SD**	**Median (min/max)**	**Mean ± SD**	**Median (min/max)**	**Mean ± SD**	**Median (min/max)**	

Age (years)	15.66 ± 3.82	16.00 (7.00/21.00)	31.76 ± 7.76	30.00 (22.00/49.00)	60.52 ± 7.40	59.50 (50.00/81.00)	**< 0.001**
ΔSD between J0_(OR) and J0_(SR)	0.10 ± 0.16	0.01 (−0.12/1.00)	0.06 ± 0.18	0.00 (−0.62/0.70)	−0.02 ± 0.17	−0.00 (−0.62/0.86)	**< 0.001**
ΔSD between J45_(OR) and J45_(SR)	−0.01 ± 0.07	0.00 (−0.32/0.25)	−0.00 ± 0.09	0.00 (−0.27/0.35)	−0.02 ± 0.09	0.00 (−0.38/0.26)	0.067
ΔAD between CP_(OR) and CP_(SR)	0.23 ± 0.33	0.00 (0.00/2.00)	0.24 ± 0.33	0.25 (0.00/1.50)	0.24 ± 0.28	0.25 (0.00/1.75)	0.188
SPD between CP_(OR) and CP_(SR)	14.27 ± 17.99	0.00 (0.00/66.67)	16.53 ± 20.71	8.00 (0.00/85.71)	18.73 ± 18.52	18.18 (0.00/66.67)	0.095
DV between OR and SR	0.22 ± 0.24	0.18 (0.00/1.42)	0.26 ± 0.30	0.18 (0.00/2.00)	0.26 ± 0.23	0.18 (0.00/1.24)	0.240
ΔAX@1D between OR and SR	7.16 ± 9.25	5.00 (0.00/57.39)	7.46 ± 9.20	7.16 (0.00/42.97)	7.68 ± 7.93	7.16 (0.00/50.41)	0.338
ΔSD between J0_(OR) and J0_(KR)	0.22 ± 0.55	0.00 (−0.76/1.94)	0.05 ± 0.53	0.00 (−1.75/1.95)	−0.17 ± 0.72	−0.16 (−3.00/2.00)	**< 0.001**
ΔSD between J45_(OR) and J45_(KR)	−0.04 ± 0.29	0.00 (−1.26/1.06)	0.04 ± 0.32	0.00 (−1.48/1.11)	−0.07 ± 0.39	−0.00 (−1.30/1.10)	**0.015**
ΔAD between CP_(OR) and CP_(KR)	0.70 ± 0.67	0.50 (0.00/2.75)	0.64 ± 0.61	0.50 (0.00/2.75)	0.63 ± 0.57	0.50 (0.00/3.50)	0.833
SPD between CP_(OR) and CP_(KR)	52.07 ± 41.14	50.00 (0.00/200.00)	50.21 ± 37.43	40.00 (0.00/150.00)	55.38 ± 42.18	50.00 (0.00/200.00)	0.764
DV between OR and KR	0.63 ± 0.68	0.35 (0.00/3.18)	0.63 ± 0.61	0.35 (0.00/3.18)	0.90 ± 0.78	0.71 (0.00/4.24)	**0.002**

*Note:* F = female, M = male, J0 and J45 = Jackson cross‐cylinder components, ΔAD = absolute differences, ΔSD = signed differences, DV = difference vector (the Euclidean distance in power vector space), ΔAX@1D = Axis deviation (°) normalized to 1.00 D of cylinder power, derived from ΔJ0 and ΔJ45, KR = values derived from keratometric readings. Data are presented as mean ± standard deviation and median (minimum–maximum) values, as appropriate. *p* values indicate between‐group comparisons using the Kruskal–Wallis test. Post hoc pairwise comparisons were conducted using Bonferroni correction. Statistical significance was defined as *p* < 0.05. Bold values indicate statistically significant results.

Abbreviations: CP, cylinder power; OR, objective refraction; SD, standard deviation; SPD, symmetric percentage difference; SR, subjective refraction.

### 3.6. Clinical Agreement Rates

Using predefined clinical thresholds (SE ≤ ±0.50 D, CP ≤ ±0.25 D, and axis deviation ≤ 5° or ≤ 10° in eyes with cylinder ≥ 0.75 D), agreement rates were high across the study population. Overall, 5.93% of eyes showed an SE difference greater than ±0.50 D, whereas 40.15% exceeded a CP difference of ±0.25 D. Axis deviations > 5° and > 10° occurred in 29.63% and 27.21% of eyes, respectively. Despite these deviations, the majority of eyes remained within clinically acceptable agreement limits. The age‐group distribution of agreement is summarized in Table 5.

**TABLE 5:** Clinical agreement rates for spherical equivalent, cylinder power, and axis alignment across age groups.

Variable	Total (%)	7–21 years	22–49 years	≥ 50 years
SE difference > ± 0.50 D	49 (5.93%)	13 (1.57%)	27 (3.26%)	9 (1.09%)
CP difference > ± 0.25 D	332 (40.15%)	87 (10.52%)	124 (14.99%)	121 (14.63%)
Axis deviation > 5°	245 (29.63%)	63 (7.62%)	86 (10.40%)	96 (11.61%)
Axis deviation > 10°	225 (27.21%)	59 (7.13%)	77 (9.31%)	89 (10.76%)

Abbreviations: CP, cylinder power; SE, spherical equivalent.

## 4. Discussion

This study provides a comprehensive assessment of the relationship between noncycloplegic autorefraction and subjective refraction across multiple age and refractive subgroups within a large real‐world clinical population. The principal observation was that differences in SE between the two approaches were consistently small and within clinically acceptable limits across all age groups, whereas subtle but systematic vector shifts were observed in astigmatic components, particularly in J0.

The overall population demonstrated a high level of concordance, with mean absolute SE differences remaining below 0.25 D. These findings are consistent with previous reports indicating that noncycloplegic autorefraction yields clinically reliable SE estimates in the presence of stable accommodation, especially in adult and myopic eyes [4–6, 13]. In myopic individuals, both SE and cylindrical agreement remained substantial across all ages, supporting the assumption that accommodative influence on myopia is limited from adolescence to older adulthood.

In hyperopic eyes, mean SE discrepancies remained ≤ 0.33 D, and more than 90% of eyes met the predefined clinical threshold of ±0.50 D. These results suggest high interchangeability between noncycloplegic autorefraction and subjective refraction during routine examinations. Previous research has emphasized that noncycloplegic autorefraction may underestimate hyperopia due to latent accommodation, particularly among children or young adults with high accommodative demand [6, 14, 15]. However, the current study excluded latent hyperopes, which likely contributed to the favorable outcomes observed in younger adults. Therefore, while the present results support the clinical utility of autorefraction in cooperative adult hyperopes, they should not be generalized to pediatric or latent hyperopic populations, in whom cycloplegia remains essential.

Analysis of astigmatic components revealed physiologically expected vector trends across age groups. The J0 vector demonstrated a gradual shift from positive values in younger participants to negative values in older adults, reflecting the well‐established transition from with‐the‐rule to against‐the‐rule astigmatism. This shift is believed to result from progressive lenticular and posterior corneal changes [11, 16]. In contrast, J45 remained close to zero across all groups, indicating minimal oblique astigmatism. Although axis deviation occurred in a considerable proportion of eyes, these deviations were frequently associated with low cylinder magnitudes, where the visual consequences are minimal. This observation underscores that axis differences alone may be misleading without considering the corresponding CP. In eyes with higher cylinder magnitudes, numerical axis differences were smaller, most likely due to more distinct subjective cues and more reliable refinement during examination.

Although previous literature has shown that even minor axis misalignment may lead to measurable degradation in optical quality [17], the majority of eyes in the present study had low astigmatic power. In such cases, reduced axis precision likely reflects measurement variability and the minimal perceptual importance of axis orientation, rather than a meaningful refractive disagreement. Consequently, vector‐based disagreement must be interpreted in the context of both cylinder magnitude and age‐related internal astigmatism. The small but systematic shift toward negative J0 values with increasing age corresponds with prior research describing the physiological progression from with‐the‐rule to against‐the‐rule astigmatism [12, 18]. In older adults and in eyes with higher cylinder values, however, greater attention to axis refinement may be warranted to achieve the best optical outcome.

Vector analysis demonstrated that discrepancies between objective and subjective refraction are influenced by both corneal and internal optical structures. In pure astigmatism, J0 and J45 differences remained ≤ 0.10 D and within clinical tolerance. These findings are consistent with earlier studies reporting high repeatability and reproducibility of power‐vector‐based subjective refraction, even under noncycloplegic conditions [9, 10, 19]. However, in cases of mixed astigmatism, J0 exhibited a progressive shift from positive to negative values with advancing age (approximately +0.10 D in the eyes of younger patients to −0.02 D in the eyes of elderly patients), indicating increasing lenticular and posterior corneal contributions to total astigmatism [12, 18]. These patterns highlight the necessity for careful axis refinement during subjective assessment in older patients and in eyes with higher CP.

Sex‐related differences in agreement were minimal. Although male participants tended to be slightly older and exhibited marginally higher DVs between total and corneal astigmatism, the magnitude of these differences was small and unlikely to affect clinical decision‐making. The observed variation is more plausibly attributable to normal biomechanical or lenticular factors rather than meaningful refractive disagreement.

Clinical agreement thresholds strengthened the evidence for practical reliability. Most eyes demonstrated acceptable SE agreement, and although axis deviations were more common, these frequently occurred in eyes with low CP, in which perceptual impact is limited. Consequently, axis deviations > 10° do not necessarily indicate clinically significant disagreement, particularly when cylinder magnitude is low. These observations suggest that axis discrepancy should be evaluated relative to cylinder strength rather than interpreted in isolation.

Several limitations should be acknowledged. The noncycloplegic design raises the possibility of residual accommodation, particularly among younger subjects, although standardized fogging and duochrome test procedures were applied. The retrospective single‐center design may influence generalizability, and biometric variables including posterior corneal astigmatism and axial length were not available. Moreover, the exclusion of latent hyperopes and pediatric hyperopia may have contributed to a more favorable agreement profile in younger groups.

Despite these limitations, the study’s strengths include its large sample size, real‐world clinical setting, and comprehensive power‐vector analysis across a broad age spectrum. The application of clinically relevant thresholds for SE, cylinder, and axis enhances the practical interpretability of the findings and supports the clinical relevance of autorefraction in routine ophthalmic practice.

## 5. Conclusion

Noncycloplegic autorefraction demonstrated strong agreement with subjective refraction for SE across adolescents and adults. Astigmatic discrepancies were generally small and of limited visual relevance, with larger axis deviations primarily occurring in eyes with low CP. These findings support the use of autorefraction as a reliable first‐line modality in routine clinical practice, while emphasizing the need for careful subjective refinement in hyperopic, mixed‐astigmatic, and the eyes of elderly patients in which internal optics exert greater influence. Furthermore, the concordance in SE indicates that noncycloplegic autorefraction may also offer practical value during the early evaluation of patients who could later be considered for refractive surgery. However, a comprehensive subjective and, when indicated, cycloplegic refraction should continue to serve as the basis for a definitive preoperative assessment.

## Funding

This research received no external funding.

## Conflicts of Interest

The authors declare no conflicts of interest.

## Data Availability

The data that support the findings of this study are available on request from the corresponding author. The data are not publicly available due to privacy or ethical restrictions.
